# Dataset in the characterization of black spot Ehrenberg snapper and its proteins' denaturation inhibition by natural antioxidants

**DOI:** 10.1016/j.dib.2019.104927

**Published:** 2019-12-05

**Authors:** Abdelaziz Elgamouz, Rana Alsaidi, Alaa Alsaidi, Mostafa Zahri, Ahmed Almehdi, Khalid Bajou

**Affiliations:** a Department of Chemistry, College of Sciences, Research Institute of Science and Engineering, University of Sharjah, P.O. Box 27272, Sharjah, United Arab Emirates; b Department of Mathematics, College of Sciences, University of Sharjah, P.O. Box 27272, Sharjah, United Arab Emirates; c Department of Applied Biology, College of Sciences, University of Sharjah, P.O. Box 27272, Sharjah, United Arab Emirates

**Keywords:** Fish conservation, Proteins'unfolding, Natural antioxidant, Thermodynamics, Polyphenols, Flavonoids

## Abstract

The data represented in this paper describe techniques, methodologies and data obtained during the biochemical composition characterization of Blackspot Snapper (*Ehrenberg's Snapper*). Data analysis of protein, lipids, moisture, ash contents of Ehrenberg's snapper, total **polyphenols**, total flavonoids contents and the DPPH scavenging activities of Cinnamon (*Cinnamomum verum J. Presl*) bark (50 mg/50 g), cumin (*Cuminum cyminum* L.) (50 mg/50 g), turmeric (*Turmerica longa* L.) (50 mg/50 g), garlic (*Allium sativum* L.) (50 mg/50 g), ginger (*Zingiber officinale Roscoe*) (50 mg/50 g) and Vitamin C (25 mg/50 g) are represented. Data obtained from the Infrared spectroscopy (FTIR) analysis of the six spices and vitamin C treated and stored fillets at −25 °C, namely three vibrations, Amide A, NH stretching at 3300 cm^−1^; Amide I, C=O stretching 1600−1690 cm^−1^ and Amide II, CN stretching and NH bending at 1480−1575 cm^−1^. Differential scanning calorimetry (DSC) analysis data of three main denaturations; myosin, actin and sarcoplasmic proteins are presented.

**Table:** Specifications Table

Subject	1.6: Agricultural and Biological Sciences: Food Science 3.2: Biochemistry, Genetics and Molecular Biology: Biochemistry 6.1: Chemistry: Analytical Chemistry
Specific subject area	Food Science and analytical Chemistry
Type of data	Table, image, graph, figure, text file
How data were acquired	UV-2510TS Single Beam Thermo Scientific Multiskan 1510–02828C spectrophotometer Bruker Platinum ATR tensor II FTIR spectrometer XGT-7200 X-ray Analytical Microscope – Horiba Differential Scanning Calorimetry, DSC-Q20 SPSS 15.0 version for windows evaluation version
Data format	Analyzed
Parameters for data collection	• Concentration of antioxidant (natural and synthetic). • FT-IR transmittance of treated and non-treated mice fillets • Time ranges from 1 week to 4 weeks. • T_m_: The melting temperature of the protein when half of the protein is folded (native) and the other half is unfolded (denatured) • ΔH⁰_U_: Specific Enthalpy change of unfolding at T_m_ (J/g)
Description of data collection	Ehrenberg's snapper fillets were minced and marinated with water extracts of antioxidants, namely: cinnamon, cumin turmeric, garlic, ginger and vitamin C. Samples were stored at −25 °C in the freezer then analyzed by FT-IR and DSC.
Data source location	Sharjah/North region/Arabian Gulf United Arab Emirates Latitude 25.3463° N, longitude 55.4209° E and GPS coordinates 25° 19′ 20.3772″ N and 55° 30′ 49.1076″ E.
Data accessibility	The data represented is with this article.
Related research article	Abdelaziz Elgamouz, Rana Alsaidi, Alaa Alsaidi, Mostafa Zahri, Ahmed Almehdi and Khalid Bajou, The effects of storage on quality and nutritional values of Ehrenberg's Snapper (Lutjanus ehrenbergi): evaluation of natural antioxidants effect on the denaturation of proteins, Journal of Biomolecules, https://doi.org/10.3390/biom9090442 [1].

**Table:** 

**Value of the Data** • The research represents a very useful data for proteins' denaturation inhibition by using natural antioxidants. • These data are relevant to food conservation, especially sustainable fish species and provide more understanding of factors affecting proteins' denaturation. • These data gave a detailed and complete set of experiments that could be used in the characterization of various fish species and provide an insight on how gels, pastes and surimi could be prepared from seafood. • The data reveal new ways in which widely used natural antioxidants presumably used to inhibit protein denaturation and develop more sustainable and innovative products from widely available stocks of fish and save overfished categories.

## 1 Data

**Fig. 1:**
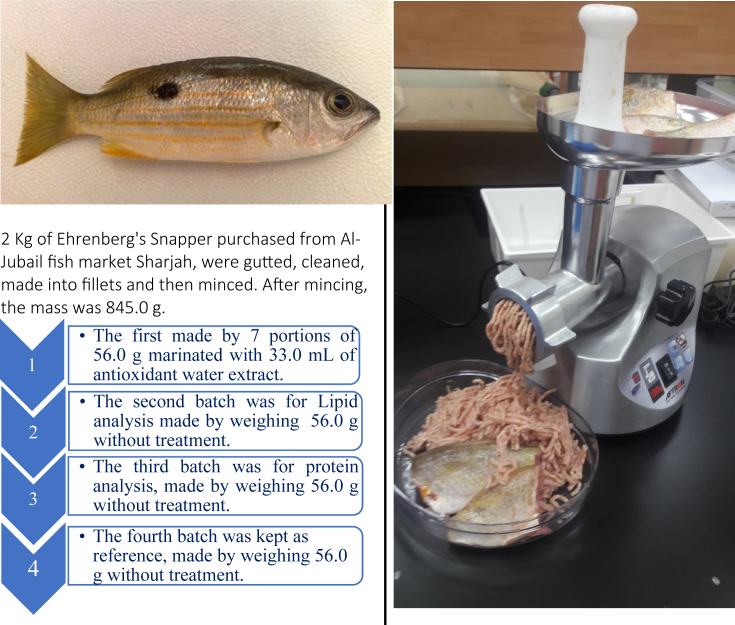
Protocol and mincer machine used for the preparation of 845.0 g of Ehrenberg snapper's mince fillets which was divided into 56.0 g boxes to make four different batches.

**Fig. 2:**
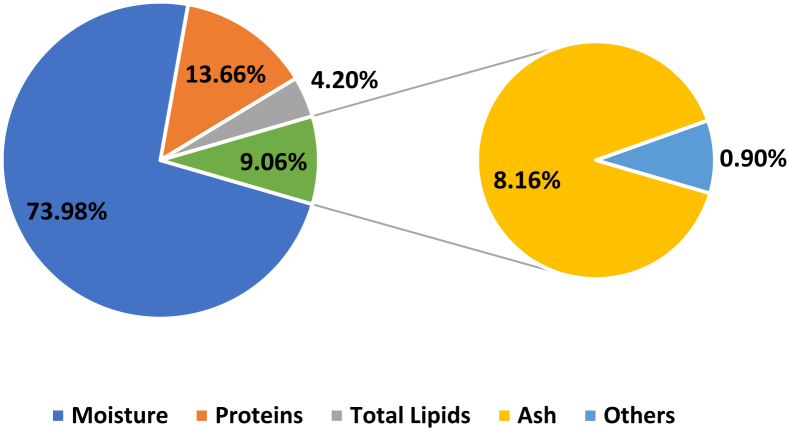
Composition of Ehrenberg's Snapper black spot fish.

**Fig. 3:**
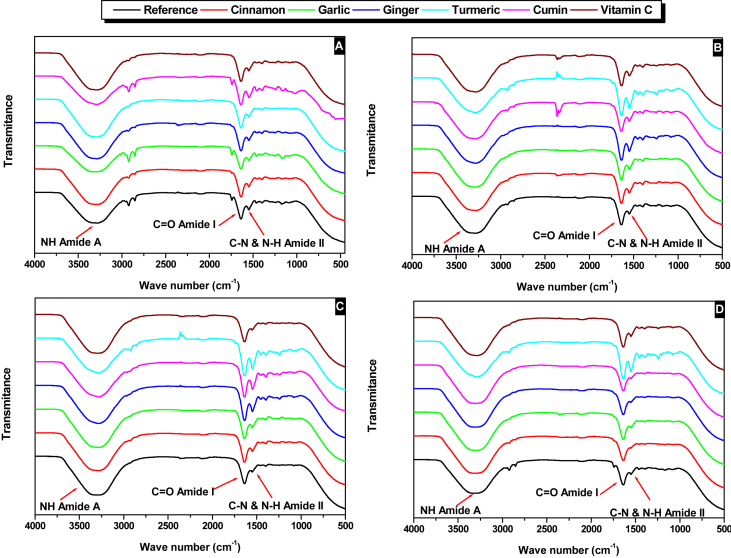
FTIR for frozen fish (−25.0 °C) Ehrenberg's Snapper treated with; 50 mg garlic; 50 mg cinnamon; 50 mg cumin; 50 mg turmeric; 50 mg garlic; 50 mg ginger; and 25.0 mg of vitamin C per 50 g of mince fillets; and without antioxidant for control after (A) 1 week, (B) 2 weeks, (C) 3 weeks and (D) 4 weeks' time.

**Fig. 4:**
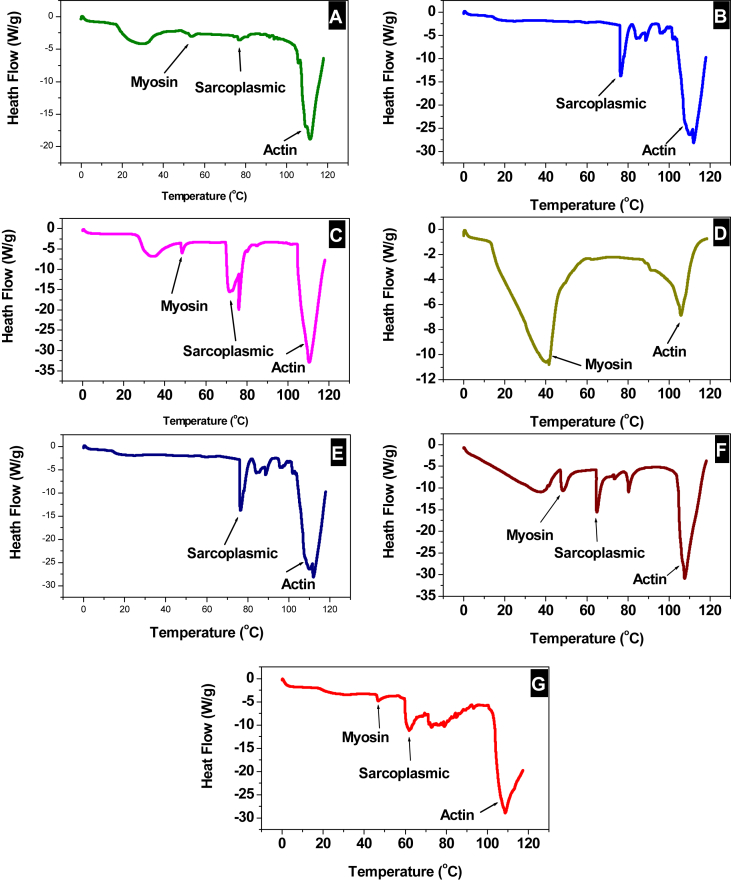
Differential scanning calorimetry of Ehrenberg snapper during storage of 30 days at −25 °C mixed with 50 mg biomass/50 g of mince fillets of; (A) cumin, (B) turmeric, (C) garlic, (D) cinnamon, (E) ginger, (F) vitamin C and (G) control without antioxidant.

Here we report experimental data on proteins' denaturation of Ehrenbergs' Snapper (*Lutjanus ehrenbergii*) locally known as (*Naiser*) which fall within the green category according to the consumer guide produced by Emirates Wildlife Society, in association with the World Wide Fund for Nature (EWS-WWF) [2]. This guide helped in the identification of more sustainable species. Calibration curves for assays for polyphenols, flavonoids contents and DPPH scavenging activity of garlic, ginger, cumin, turmeric and cinnamon are presented in (Figure S1, S2 and S3 respectively, included in Supplementary documents). While data, of total phenolic contents presented in mg (gallic acid)/100 g (dry weight of biomass), flavonoids presented as ppm of rutin and DPPH scavenging activity given in inhibition (%), are given in Table 1. Fish samples were prepared using protocol shown in Fig. 1. The biochemical characterization of Ehrenberg snapper includes; lipids content, peroxide value, moisture content, ash analysis, proteins content are presented in Fig. 2. The metal content of ash are represented in Table 2. The effect of 50.0 mg biomass/50 g mince fillets treated with cinnamon, cumin, turmeric, garlic, ginger and 25.0 mg of vitamin C was studied to assess protein denaturation during a period of 4 weeks storage time at −25.0 °C. FT-IR stretching vibration of Amide-A (ν_NH_) at 3300 cm^−1^; Amide-I stretching (ν_C_=_O_) between 1600 and 1690 cm^−1^ and Amide-II stretching (ν_CN_) and bending (δ_NH_) between 1480 and 1575 cm^−1^ were used as marker peaks, these are presented from Fig. 3. Averages of transmittances of the marker peaks run in triplicate for different antioxidant treated and non-treated samples frozen at −25 °C for 1 week, 2 weeks, 3 weeks and 4-weeks storage time were summarized in Table 3. Descriptive analysis one-way ANOVA and pair-wise comparison of mean values of different variables obtained from Table 3 and summarized in Table S1 (in Supplementary material) were performed using SPSS 15.0 version, the *t*-test were used with a significant level of p < 0.05. The data are presented from Table S2 to Table S3 (Supplementary material). In table S1, is presented the statistical coding of different variable. For instance, the outputs of the first week, Amide A is denoted by V13A1, V13A2 V13A3, V13A4, V13A5, V13A6, V13A7, respectively, where 13 denotes first week and numbers from 1 to 7 denote antioxidants; reference, cinnamon, garlic, ginger, turmeric, cumin and vitamin respectively. Similar coding was used for other weeks. Comparison was made in two different ways; a horizontal comparison to assess the effect of antioxidant, the *t*-test in this case was performed with a reference variable V13A1 and all other variables V13A2 V13A3 V13A4 V13A5 V13A6 V13A7, the code used in SPSS to calculate p values in this case is given as Code 1. While the vertical comparison was made to assess the effect of time, in this case comparison was made through the same categories; amide A (week-1) with amide A (week-2). The code used in this case is presented as Code 2. We finally analyzed the effect of 50.0 mg biomass/50 g mince fillets treated with cinnamon, cumin, turmeric, garlic, ginger and 25.0 mg of vitamin C on protein denaturation during a period of 4 weeks and storage time of −25.0 °C using DSC, three marker peaks were followed, myosin, sarcoplasmic proteins and actin. Thermograms are represented in Fig. 4, peaks (°C) and enthalpies (J/g) obtained for different antioxidants treated mince fillets from DSC are summarized in Table 4.Code 1
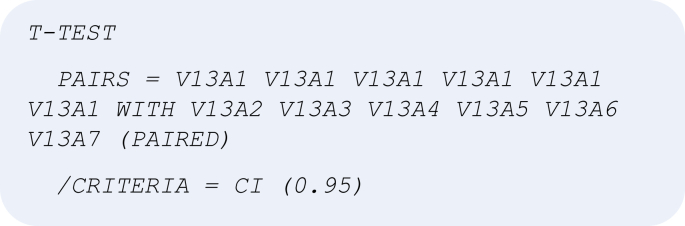
Code 2
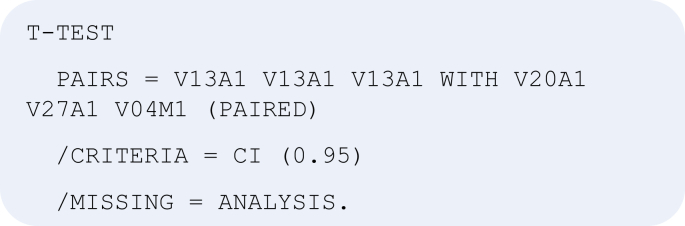

**Table 1:** Total phenolic contents in mg (GAE)/100 g (DW), total flavonoids contents and DPPH radical scavenging activity of garlic, ginger, cumin, turmeric, and cinnamon.

Antioxidant	mg (GAE)/100 DW	DPPH (inhibition) %	10. mg/mL	1.0 mg/mL	0.5 mg/mL
Garlic	3.73 ± 0.01	89.87%	26.13 ± 0.01	1.80 ± 0.001	0.58 ± 0.31
Ginger	5.92 ± 0.02	80.45%	62.04 ± 0.02	4.28 ± 0.01	1.39 ± 0.07
Cumin	13.26 ± 0.01	7.086%	129.46 ± 0.05	8.93 ± 0.01	2.89 ± 0.15
Turmeric	20.34 ± 0.01	90.50%	70.85 ± 0.03	4.89 ± 0.01	1.58 ± 0.08
Cinnamon	37.93 ± 0.19	90.54%	44.51 ± 0.02	3.07 ± 0.001	0.99 ± 0 .05

**Table 2:** Metal composition of Ehrenberg's snapper's analyzed by X-ray fluorescence (XRF).

Elem.	Line	Mass [%]	3 sigma	Atomic [%]	Intensity [cps/mA]	mg/Kg
12 Mg	K	1.42	0.27	2.13	11.99	1159.67
15 P	K	24.06	0.11	28.28	2468.81	19649.00
16 S	K	3.23	0.03	3.66	403.94	2637.83
17 Cl	K	2.38	0.03	2.44	298.5	1943.67
19 K	K	54.89	0.18	51.1	6343.13	44826.83
20 Ca	K	13.09	0.1	11.89	953.49	10690.17
26 Fe	K	0.31	0.01	0.2	99.28	253.17
30 Zn	K	0.21	0.01	0.12	153.62	171.50
38 Sr	K	0.41	0.01	0.17	583.24	334.83

**Table 3:** Average transmittance of antioxidant treated and non-treated samples and frozen at −25 °C at 1 week, 2 weeks, 3 weeks and 4-weeks storage timing.

Date	Peak type	Reference		Cinnamon		Garlic		Ginger		Turmeric		Cumin		Vitamin C	
		ν(cm^−1^)	Trans	ν(cm^−1^)	Trans	ν(cm^−1^)	Trans	ν(cm^−1^)	Trans	ν(cm^−1^)	Trans	ν(cm^−1^)	Trans	ν(cm^−1^)	Trans
Week-1	Amide A 3300 NH stretch	3299.944	0.50538	3299.944	0.46547	3299.944	0.57851	3299.944	0.46575	3299.944	0.59054	3299.944	0.48834	3299.944	0.48438
			0.51138		0.53147		0.65251		0.52275		0.62354		0.50334		0.51138
			0.51738		0.59747		0.72651		0.57975		0.65654		0.51834		0.53838
			±0.006		±0.066		±0.074		±0.057		±0.033		±0.015		±0.027
	Amide I 1600–1690 C=O stretch	1634.953	0.56936	1634.953	0.59969	1643.535	0.64828	1642.105	0.57338	1634.953	0.58004	1642.105	0.61003	1634.953	0.57136
			0.62236		0.62869		0.69028		0.62538		0.61904		0.62103		0.62236
			0.67536		0.65769		0.64828		0.67738		0.65804		0.63203		0.67336
			±0.053		±0.029		±0.042		±0.052		±0.039		±0.006		±0.051
	Amide II 1480–1575 CN stretch NH bending	1549.128	0.72931	1549.128	0.73186	1549.128	0.78289	1549.128	0.75324	1547.698	0.66201	1549.128	0.73206	1549.128	0.80731
			0.75731		0.76186		0.80089		0.75724		0.71101		0.75906		0.75731
			0.78531		0.79186		0.81889		0.76124		0.76001		0.78606		0.80731
			±0.028		±0.003		±0.018		±0.004		±0.049		±0.027		±0.005
Week-2	Amide A 3300 NH stretch	3299.943	0.47734	3299.944	0.49232	3299.944	0.48294	3299.94	0.47704	3299.944	0.5124	3299.944	0.50352	3299.944	0.48852
			0.52234		0.51032		0.50694		0.51104		0.5514		0.52152		0.51552
			0.56734		0.52832		0.53094		0.54504		0.5904		0.53952		0.54252
			±0.045		±0.018		±0.024		±0.034		±0.039		±0.018		±0.027
	Amide I 1600–1690 C=O stretch	1634.952	0.59334	1634.953	0.56349	1634.953	0.58094	1634.953	0.5283	1633.522	0.46895	1634.953	0.58063	1634.953	0.55223
			0.62934		0.60149		0.59794		0.5493		0.51295		0.61363		0.59223
			0.66534		0.63949		0.61494		0.5703		0.55695		0.64663		0.63223
			±0.036		±0.038		±0.017		±0.021		±0.044		±0.033		±0.040
	Amide II 1480–1575 CN stretch, NH bending	1549.128	0.69048	1549.128	0.682	1547.698	0.70426	1547.698	0.60891	1547.698	0.59117	1549.128	0.70683	1547.698	0.70802
			0.76548		0.732		0.72826		0.66191		0.60217		0.74283		0.71702
			0.84048		0.782		0.75226		0.71491		0.61317		0.77883		0.72602
			±0.075		±0.05		±0.024		±0.053		±0.011		±0.036		±0.009
Week-3	Amide A 3300 NH stretching	3299.943	0.49085	2999.55	0.78459	3299.944	0.43806	3299.944	0.49528	3299.944	0.4808	3301.374	0.45346	3299.944	0.47885
			0.50085		0.86159		0.50606		0.50628		0.5488		0.50846		0.50085
			0.51085		0.93859		0.57406		0.51728		0.6168		0.56346		0.52285
			±0.01		±0.077		±0.068		±0.011		±0.068		±0.055		±0.022
	Amide I 1600–1690 C=O stretch	1634.952	0.55516	1634.953	0.53463	1634.953	0.53885	1634.953	0.53669	1634.953	0.48196	1634.953	0.54212	1634.953	0.62116
			0.64616		0.61863		0.61585		0.62569		0.54496		0.63812		0.64616
			0.73716		0.70263		0.69285		0.71469		0.60796		0.73412		0.67116
			±0.091		±0.084		±0.077		±0.089		±0.063		±0.096		±0.022
	Amide II 1480–1575 CN stretch NH bending	1634.953	0.60716	1549.128	0.67242	1549.128	0.60229	1549.128	0.68625	1547.698	0.52599	1549.128	0.73035	1550.559	0.74912
			0.64616		0.75642		0.75529		0.76725		0.64199		0.78135		0.79512
			0.68516		0.84042		0.90829		0.84825		0.65359		0.83235		0.84112
			±0.039		±0.084		±0.153		±0.081		±0.0116		±0.051		±0.046
Week-4	Amide A 3300 NH stretch	2999.558	0.8337	3299.944	0.45725	3299.944	0.41108	3299.944	0.45225	2999.559	0.78016	3299.944	0.46918	2999.559	0.77868
			0.8727		0.51725		0.50808		0.50325		0.85116		0.50418		0.86068
			0.9117		0.57725		0.60508		0.55425		0.92216		0.53918		0.94168
			±0.039		±0.060		±0.097		±0.051		±0.071		±0.035		±0.081
	Amide I 1600–1690 C=O stretch	1634.953	0.65331	1634.953	0.66455	1634.953	0.61647	1634.953	0.65523	1633.52	0.49238	1634.953	0.64657	1633.522	0.58776
			0.67031		0.67955		0.64747		0.66223		0.51338		0.66357		0.61976
			0.68731		0.69455		0.67847		0.66923		0.53438		0.68057		0.65176
			±0.017		±0.022		±0.022		±0.007		±0.021		±0.017		±0.032
	Amide II 1480–1575 CN stretch, NH bending	1549.128	0.79449	1574.876	0.75328	1549.128	0.68594	1550.559	0.74015	1550.559	0.7023	1550.559	0.74433	1549.128	0.30511
			0.80549		0.83328		0.79594		0.81715		0.6063		0.81833		0.75511
			0.81649		0.91328		0.90594		0.89415		0.7023		0.89233		0.80011
			±0.011		±0.080		±0.11		±0.077		±0.096		±0.074		±0.045

**Table 4:** DSC thermograms summary for frozen Ehrenberg's snapper with and without antioxidants at −25 °C.

Sample	Tm (°C) myosin peak	ΔH⁰_U_ (J/g) myosin peak	Tm (°C) actin peak	ΔH⁰_U_ (J/g) actin peak
Reference	46.87	5.949	108.59	200.9
Frozen Turmeric sample	75.51	73.69	111.97	482.8
Frozen garlic sample	48.45	7.168	110.32	431.2
Frozen cinnamon sample	41.44	769.3	105.79	239.0
Frozen cumin sample	28.17	91.24	111.37	248
Frozen ginger sample	No peak	No peak	102.01	1367
Frozen Vitamin C sample	48.30	28.65	107.61	340.5

## 2 Experimental design, materials, and methods

### 2.1 Natural antioxidant extraction

Natural antioxidants, cinnamon sticks, cumin seeds, ginger powder, turmeric powder and fresh garlic paste were purchased from the local market of Aljubail, Sharjah, the United Arab Emirates. Cinnamon sticks, cumin seeds were grided while fresh garlic was crushed in a mortar to get a past. 5.0 g of each spice were extracted with 100 mL of water or methanol in 250 mL conical flasks which were left in horizontal mechanical shaker for a period of 2 hours at 80 °C. The spice extract were filtered on an 11.0 μm pore size Whatman filter, the filtrates were dried on a rotary evaporator and 150 mg obtained powders were dissolved in 100 mL of water or methanol and transferred to amber bottles and stored in the fridge for further analysis. 50 mg of vitamin C however were dissolved in water or methanol were used as synthetic antioxidant reference. Methanol extracts were used for tests of total polyphenols analysis, total flavonoids analysis and DPPH scavenging activity, while water extracts were used for the marinating of the fish sample to study the effect of antioxidant on the protein denaturation inhibition in Ehrenberg's snapper.

### 2.2 Total phenolic contents

Total phenolic contents in garlic, ginger, cumin, turmeric and cinnamon were determined by using the protocol described in Ref. [3]. 1.00 mL of natural antioxidant (spice) containing 1.00 mg/mL of dray mass of spice were mixed with 1.0 mL of Folin-Ciocalteu's phenol. The solution was incubated for 5 min at 23.0 °C, then 10.0 mL of a 7.00 (m/V)% sodium carbonates (Na_2_CO_3_) solution were added to the mixture. 13.0 mL of deionised water was added to the mixture to diluted it and was shacked with the rotamixer for a period of 1.0 min. The reaction mixture was kept in the dark at a temperature of 23.0 °C for 90 minutes then absorbances were measured at 750 nm using a spectrophotometer (UV-2510TS–Labomed). The same procedure was followed for the standard of pure gallic acid with concentration ranging from 25.0 to 400 mg/L. Results were expressed in mg of gallic acid equivalent per 100 g of sample dry mass (mg (GAE)/100 g DW).

### 2.3 Total flavonoids content

Rutin was used to construct the calibration curve to measure the contents of spices in flavonoids, a standard curve of rutin in the range of 10.0–80.0 ppm was prepared from 400 ppm stock solution. Flavonoids were measured according to the method described by Ref. [4]. 0.300 mL of each methanolic extract (stock solution, 150.0 mg/100 mL) of natural antioxidant (spice) were introduced into In 10.0 mL test tubes and were mixed separately with 3.40 mL of 30.0 (v/v)% methanol, 0.15 mL of 0.50 M sodium nitrate (NaNO_2_) and 0.15 mL of 0.30 M aluminium chloride hexahydrate (AlCl_3_.6H_2_O), the mixture was incubated at 23.0 °C for a period of 5 min. 1.00 mL of 1.0 M sodium hydroxide (NaOH) was added to the mixture. A blank was prepared by mixing the same reagents without any antioxidant extracts. Sample solutions and standards were homogenized, then absorbances were measured at 356 nm using a UV-VIS Spectrophotometer (UV-2510TS–Labomed).

### 2.4 DPPH scavenging activity

DPPH radical was used to assess the scavenging activity of total polyphenols present in natural antioxidants as well as vitamin C. The test performed using the protocol described in Ref. [5].

In summary, in 5 test tubes containing 2.50 mL of mathanolic extract of natural antioxidant (stock solution 150 mg/100 mL), 2.00 mL of 0.50 mM of 2,2-diphenyl-1-picrylhydrazyl (DPPH) freshly prepared in methanol were added. The mixtures were incubated at 23.0 °C for a period of 30 minutes to allow reactions to take place. The UV–Vis absorbances were measured at a wavenumber of 517 nm using a UV-VIS Spectrophotometer (UV-2510TS–Labomed). Methanol was used as a blank. Absorbances of stock solutions represent the control absorbance (A_before_) of the test and $A_{after}$ is the test's absorbance.

### 2.5 Lipid extraction

In the thimble of soxhlet apparatus mounted on a around bottle flask containing 90.0 mL of petroleum ether on the top of a heating mantle, 10.0 g of Ehrenberg's snapper's freshly prepared mince fillets. The mince fillets were extracted in the apparatus for a period of 3 hours, then, the extraction solvent was evaporated in the rotary evaporator. The mass of oil obtained was measured on analytical balance.

### 2.6 Peroxide value (PV)

Primary oxidation of Ehrenberg snapper's oil and the measurement of hydroperoxides were determined by peroxide value analysis, that consist of measuring the amount of iodine formed by the reaction of peroxides formed in fat or oil with iodide ion. The test was performed using protocol described by Ref. [6]. In 250 mL conical flask containing 10.0 mL of chloroform (CHCl_3_), 15.0 mL of glacial acetic acid (CH_3_COOH) and 1.00 mL of freshly prepared potassium iodide (KI), 0.18 g of fish oil extracted by soxhlet apparatus. The conical flask was tightly closed and gently swirled to allow its contents to mix for 1 min and kept for another 1 min in the dark. 1.00 mL of starch solution (2.00% m/v) and 75.0 mL distilled water were added to the mixture. The solution was titrated with 0.01 M sodium thiosulfate (Na_2_S_2_O_3_). The indicator was added towards the end of the titration while the pale straw colour is still present. The solution was shaken during titration until the blue colour disappeared. A blank titration was carried out under the same conditions on a mixture containing all reagents used in the test except the oil. No more than 0.50 mL of 0.01 M sodium thiosulfate solution should be consumed for this purpose.

### 2.7 Proteins content

Proteins content measurement was carried based on the concept that the amino acids building blocks of protein when digested will be converted to ammonia. The test was carried out using the Kjeldahl procedure described in Ref. [7]. 1.00 g of the minced fillets was digested in 20 mL of (H_2_SO_4_, 96%) together with two selenium catalyst tablets (5.0 g K_2_SO_4_; 0.15 g CuSO_4_.5H_2_O; 0.15 g TiO_2_). The mixture was boiled in a distillation apparatus for 2 hours. The digestion of the minced fillets continued until a clear solution was developed. Then the flask was left to cool down for 15 minutes. This technique is based on the conversion of nitrogen present in proteins to ammonia in the form of ammonium sulphate. 20.0 mL of 0.50 M sodium hydroxide (NaOH) was added to allow the release of ammonia via steam distillation in the distillation apparatus, and the distillate was collected over 25.0 mL of boric acid (4.00% m/v) then titrated against a standard solution of 0.05 M sodium carbonates (Na_2_CO_3_) using methyl orange as indicator.

### 2.8 Moisture content

5.00 g of fresh fish tissue were placed in three pre-weight glass watch, then heated in an oven pre-set in 100 °C. The glass watch were kept in the oven for a period of 24 hours, then taken and cooled in a desiccator. The moisture content was determined form the mass difference between empty and desiccator dried glass watch.

### 2.9 Ash content and X-ray fluorescence (XRF) analysis

6.00 g of fresh fish tissue were placed in three pre-weight silica dishes. The dishes were heated on a hot plate under the fume hood for a period of 10 min until moisture was removed completely, then they were placed in a programmed muffle furnace set at final temperature of 550 °C with a speed of 10 °C/min, the samples were kept at this final for a period of seven hours. Ash obtained in the silica dishes were taken measured and taken to XRF analysis to determine the fish composition in metals to look for any toxic heavy metals. The XRF machine was (XGT-7200 X-ray Analytical Microscope – Horiba).

## References

[1] Elgamouz A., Alsaidi R., Alsaidi A., Zahri M., Almehdi A., Bajou K. (2019). The effects of storage on quality and nutritional values of Ehrenberg's snapper muscles (Lutjanus Ehrenbergi): evaluation of natural antioxidants effect on the denaturation of proteins. Biomolecules.

[2] Burning issue: a fishy tale. https://www.caterermiddleeast.com/18627-burning-issue-a-fishy-tale.

[3] Singleton V.L., Rossi J.A. (1965). Colorimetry of total phenolics with phosphomolybdic-phosphotungstic acid reagents. Am. J. Enol. Vitic..

[4] Park H., Lee S., Son H., Park S., Kim M., Choi E., Singh T.S., Ha J., Lee M., Kim J. (2008). Flavonoids inhibit histamine release and expression of proinflammatory cytokines in mast cells. Arch Pharm Res.

[5] Brand-Williams W., Cuvelier M., Berset C. (1995). Use of a free radical method to evaluate antioxidant activity. LWT-Food Sci. Technol..

[6] AOCS method D Firestone (1989). Official Methods and Recommendation Practices of the American Oil Chemists' Society.

[7] (1995). AOAC Methods Official Methods of Analysis of AOAC International.

