# Supplementary material for: Dataset in the characterization of black spot Ehrenberg snapper and its proteins' denaturation inhibition by natural antioxidants
Source: Data Brief. 2019 Dec 5;28:104927. doi: 10.1016/j.dib.2019.104927 (PMC6920340; doi:10.1016/j.dib.2019.104927)
Supplement: Multimedia component 1 [file mmc1.docx]

**Figure S1** Galllic acid calibration curve for total phenolic contents measured at 750 nm

**Figure S2** Rutin Calibration Curve for total flavonoids content at 356.0 nm


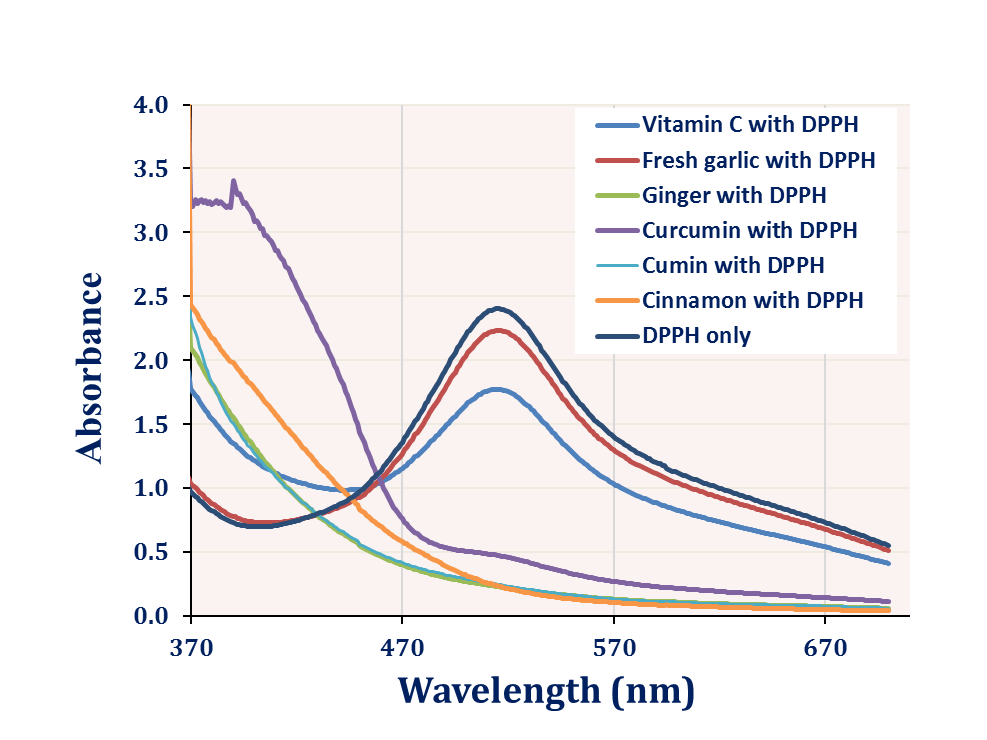


**Figure S3** UV-vis spectra of DPPH radical alone and with different antioxidants (natural antioxidants and vitamin C as synthetic antioxidant)

Table. S1 Statistical coding of different variables

|  |  | **Reference** | **Cinnamon** | **Garlic** | **Ginger** | **Turmericin** | **Cumin** | **Vitamin C** |
| --- | --- | --- | --- | --- | --- | --- | --- | --- |
|  |  | **V13A1** | **V13A2** | **V13A3** | **V13A4** | **V13A5** | **V13A6** | **V13A7** |
| **Week-1** | **Amide A** | 0.50538 | 0.46547 | 0.57851 | 0.46575 | 0.59054 | 0.48834 | 0.48438 |
|  |  | 0.51138 | 0.53147 | 0.65251 | 0.52275 | 0.62354 | 0.50334 | 0.51138 |
|  |  | 0.51738 | 0.59747 | 0.72651 | 0.57975 | 0.65654 | 0.51834 | 0.53838 |
|  |  | **V13AI1** | **V13AI2** | **V13AI3** | **V13AI4** | **V13AI5** | **V13AI6** | **V13AI7** |
|  | **Amide I** | 0.56936 | 0.59969 | 0.64828 | 0.57338 | 0.58004 | 0.61003 | 0.57136 |
|  |  | 0.62869 | 0.62103 | 0.62236 | 0.62236 | 0.69028 | 0.62538 | 0.61904 |
|  |  | 0.65769 | 0.64828 | 0.63203 | 0.67336 | 0.67738 | 0.65804 | 0.67536 |
|  |  | **V13AII1** | **V13AII2** | **V13AII3** | **V13AII4** | **V13AII5** | **V13AII6** | **V13AII7** |
|  | **Amide II** | 0.72931 | 0.73186 | 0.78289 | 0.75324 | 0.66201 | 0.73206 | 0.80731 |
|  |  | 0.71101 | 0.75906 | 0.75731 | 0.75731 | 0.76186 | 0.80089 | 0.75724 |
|  |  | 0.76001 | 0.78606 | 0.78531 | 0.79186 | 0.80731 | 0.76124 | 0.81889 |
|  |  | **V20A1** | **V20A2** | **V20A3** | **V20A4** | **V20A5** | **V20A6** | **V20A7** |
| **Week-2** | **Amide A** | 0.47734 | 0.49232 | 0.48294 | 0.47704 | 0.5124 | 0.50352 | 0.48852 |
|  |  | 0.52234 | 0.51032 | 0.50694 | 0.51104 | 0.5514 | 0.52152 | 0.51552 |
|  |  | 0.56734 | 0.52832 | 0.53094 | 0.54504 | 0.5904 | 0.53952 | 0.54252 |
|  |  | **V20AI1** | **V20AI2** | **V20AI3** | **V20AI4** | **V20AI5** | **V20AI6** | **V20AI7** |
|  | **Amide I** | 0.59334 | 0.56349 | 0.58094 | 0.5283 | 0.46895 | 0.58063 | 0.55223 |
|  |  | 0.60149 | 0.59794 | 0.5493 | 0.51295 | 0.61363 | 0.59223 | 0.62934 |
|  |  | 0.5703 | 0.64663 | 0.63223 | 0.66534 | 0.63949 | 0.61494 | 0.55695 |
|  |  | **V20AII1** | **V20AII2** | **V20AII3** | **V20AII4** | **V20AII5** | **V20AII6** | **V20AII7** |
|  | **Amide II** | 0.69048 | 0.682 | 0.70426 | 0.60891 | 0.59117 | 0.70683 | 0.70802 |
|  |  | 0.732 | 0.72826 | 0.66191 | 0.60217 | 0.74283 | 0.71702 | 0.76548 |
|  |  | 0.77883 | 0.72602 | 0.782 | 0.61317 | 0.71491 | 0.75226 | 0.84048 |
|  |  | **V27A1** | **V27A2** | **V27A3** | **V27A4** | **V27A5** | **V27A6** | **V27A7** |
| **Week-3** | **Amide A** | 0.49085 | 0.78459 | 0.43806 | 0.49528 | 0.4808 | 0.45346 | 0.47885 |
|  |  | 0.50085 | 0.50085 | 0.86159 | 0.50606 | 0.50628 | 0.5488 | 0.50846 |
|  |  | 0.52285 | 0.51085 | 0.57406 | 0.51728 | 0.6168 | 0.56346 | 0.93859 |
|  |  | **V27AI1** | **V27AI2** | **V27AI3** | **V27AI4** | **V27AI5** | **V27AI6** | **V27AI7** |
|  | **Amide I** | 0.55516 | 0.53463 | 0.53885 | 0.53669 | 0.48196 | 0.54212 | 0.62116 |
|  |  | 0.64616 | 0.61863 | 0.61585 | 0.62569 | 0.54496 | 0.64616 | 0.63812 |
|  |  | 0.69285 | 0.67116 | 0.73412 | 0.60796 | 0.71469 | 0.73716 | 0.70263 |
|  |  | **V27AII1** | **V27AII2** | **V27AII3** | **V27AII4** | **V27AII5** | **V27AII6** | **V27AII7** |
|  | **Amide II** | 0.60716 | 0.67242 | 0.60229 | 0.68625 | 0.52599 | 0.73035 | 0.74912 |
|  |  | 0.75642 | 0.79512 | 0.78135 | 0.75529 | 0.76725 | 0.64199 | 0.84112 |
|  |  | 0.83235 | 0.64616 | 0.84825 | 0.65359 | 0.84042 | 0.90829 | 0.68516 |
|  |  | **V4MA1** | **V4MA2** | **V4MA3** | **V4MA4** | **V4MA5** | **V4MA6** | **V4MA7** |
| **Week-4** | **Amide A** | 0.8337 | 0.45725 | 0.41108 | 0.45225 | 0.78016 | 0.46918 | 0.77868 |
|  |  | 0.50808 | 0.50325 | 0.85116 | 0.50418 | 0.86068 | 0.51725 | 0.53918 |
|  |  | 0.94168 | 0.8727 | 0.60508 | 0.92216 | 0.57725 | 0.55425 | 0.9117 |
|  |  | **V4MAI1** | **V4MAI2** | **V4MAI3** | **V4MAI4** | **V4MAI5** | **V4MAI6** | **V4MAI7** |
|  | **Amide I** | 0.65331 | 0.66455 | 0.61647 | 0.65523 | 0.49238 | 0.64657 | 0.58776 |
|  |  | 0.67955 | 0.64747 | 0.66223 | 0.51338 | 0.66357 | 0.61976 | 0.67031 |
|  |  | 0.67847 | 0.66923 | 0.69455 | 0.53438 | 0.68057 | 0.65176 | 0.68731 |
|  |  | **V4MAII1** | **V4MAII2** | **V4MAII3** | **V4MAII4** | **V4MAII5** | **V4MAII6** | **V4MAII7** |
|  | **Amide II** | 0.79449 | 0.75328 | 0.68594 | 0.74015 | 0.7023 | 0.74433 | 0.30511 |
|  |  | 0.80549 | 0.83328 | 0.79594 | 0.81715 | 0.6063 | 0.81833 | 0.75511 |
|  |  | 0.80011 | 0.89233 | 0.7023 | 0.81649 | 0.91328 | 0.90594 | 0.89415 |

Table. S2 Significance of amide A using the p-value between the variables using different antioxidants

| **Pairs** | | **95% confidence interval** | | **T-value** | **Two-side p-value** |
| --- | --- | --- | --- | --- | --- |
| **Pair 1** | **V13A1 – V13A2** | -0.16913826 | 0.12895826 | -0.580 | 0.621 |
| **Pair 2** | **V13A1 – V13A3** | -0.31005136 | 0.02779136 | -3.595 | 0.069 |
| **Pair 3** | **V13A1 – V13A4** | -0.13806102 | 0.11532102 | -0.386 | 0.737 |
| **Pair 4** | **V13A1 – V13A5** | -0.17923172 | -.04508828 | -7.195 | **0.019** |
| **Pair 5** | **V13A1 – V13A6** | -0.01431724 | 0.03039724 | 1.547 | 0.262 |
| **Pair 6** | **V13A1 – V13A7** | -0.05216689 | 0.05216689 | 0.000 | 1.000 |

Table. S3 Significance of amide A using the p-value between the variables at different timing (week-1 to week-4)

| **Pairs** | | **95% confidence interval** | | **T-value** | **Two-side p-value** |
| --- | --- | --- | --- | --- | --- |
| **Pair 1** | **V13A1 – V20A1** | -0.10784137 | 0.08592137 | -0.487 | 0.675 |
| **Pair 2** | **V13A1 – V27A1** | -0.01975964 | 0.03281964 | 1.069 | 0.397 |
| **Pair 3** | **V13A1 – V04M1** | -0.80711566 | 0.30756899 | -1.928 | 0.194 |

Table. S4 Significance of amide II using the p-value between the variables at different timing (week-1 to week-4)

| **Pairs** | | **95% confidence interval** | | **T-value** | **Two-side p-value** |
| --- | --- | --- | --- | --- | --- |
| **Pair 1** | **V13II1 –**  **V20II1** | -0.08460854 | 0.08395521 | -0.017 | 0.988 |
| **Pair 2** | **V13II1 –**  **V27II1** | -0.26030837 | 0.26324170 | 0.024 | 0.983 |
| **Pair 3** | **V13II1 –**  **V04II1** | -0.13419813 | 0.00102480 | -4.237 | **0.051** |

Table. S5 Significant values of Amide A, 3300cm^-1^ NH stretching for frozen samples stored for a period of 1-week.

| **Pairs** | **95% confidence interval**  **Lower-Upper** | | **T-value** | **Two-side p-value** |
| --- | --- | --- | --- | --- |
| **V13A(Ref) - V13(Turm)** | -0.17923172 | -0.04508828 | -7.195 | 0.019 |
| **V13(cinn - V13Garl)** | -0.14091310 | -0.10116690 | -26.206 | 00.01 |
| **V13(Cinn - V13Turm)** | -0.17404654 | -0.01009346 | -4.832 | 0.040 |
| **V13(Gar - V13Cin)** | 0.10116690 | 0.14091310 | 26.206 | 0.001 |
| **V13(Gar - V13Gin)** | 0.08752966 | 0.17199034 | 13.221 | 0.006 |
| **V13(Gar - V13Cur)** | 0.00260588 | 0.29573412 | 4.379 | 0.048 |
| **V13(Gar - V13Vtc)** | 0.02437553 | 0.25788447 | 5.201 | 0.035 |
| **V13(Gin - V13Garl)** | -0.17199034 | -0.08752966 | -13.221 | 0.006 |
| **V13(Gin - V13Turm)** | -0.16040931 | -0.04117069 | -7.274 | 0.018 |
| **V13(Cur - V13Gin)** | 0.04117069 | 0.16040931 | 7.274 | 0.018 |
| **V13(Cur - V13Cum)** | 0.07548552 | 0.16491448 | 11.566 | 0.007 |
| **V13(Cur - V13Vtc)** | 0.09725517 | 0.12706483 | 32.378 | 0.001 |
| **V13(Cum - V13Turm)** | -0.16491448 | -0.07548552 | -11.566 | 0.007 |
| **V13(Vtc - V13Garl)** | -0.25788447 | -0.02437553 | -5.201 | 0.035 |
| **V13(Vtc - V13Turm)** | -0.12706483 | -0.09725517 | -32.378 | 0.001 |

Table. S6 Significant values of Amide A, 3300cm^-1^ NH stretching for frozen samples stored for a period of 2-week.

| **Pairs** | **95% confidence interval**  **Lower-Upper** | | **T-value** | **Two-side p-value** |
| --- | --- | --- | --- | --- |
| **V20(Ref - V20Cur)** | -0.04396483 | -0.01415517 | -8.389 | 0.014 |
| **V20(Gin - V20Turm)** | -0.05278069 | -0.02793931 | -13.981 | 0.005 |
| **V20(Turm - V20Ref)** | 0.01415517 | 0.04396483 | 8.389 | 0.014 |
| **V20(Turm - V20Cin)** | -0.01108689 | 0.09324689 | 3.388 | 0.077 |
| **V20(Turm - V20Ging)** | 0.02793931 | 0.05278069 | 13.981 | 0.005 |
| **V20(Turm - V20Vtc)** | 0.00607035 | 0.06568965 | 5.179 | 0.035 |
| **V20(Cum - V20Garl)** | -0.00032483 | 0.02948483 | 4.209 | 0.052 |
| **V20(Vtc - V20Gar)** | 0.00112759 | 0.01603241 | 4.954 | 0.038 |
| **V20(Vtc - V20Turm)** | -0.06568965 | -0.00607035 | -5.179 | 0.035 |

Table. S7 Significant values of Amide I, 1600-1690 cm^-1^ C=O stretching for frozen samples stored for a period of 1-week.

| **Pairs** | **95% confidence interval**  **Lower-Upper** | | **T-value** | **Two-side p-value** |
| --- | --- | --- | --- | --- |
| **V13(Gal - V13Cum)** | -0.01635672 | 0.00005672 | -4.273 | 0.051 |
| **V13(Cum - V13Cin)** | -0.00005672 | 0.01635672 | 4.273 | 0.051 |

Table. S6 Significant values of Amide I, 1600-1690 cm^-1^ C=O stretching for frozen samples stored for a period of 2-week.

| **Pairs** | **95% confidence interval**  **Lower-Upper** | | **T-value** | **Two-side p-value** |
| --- | --- | --- | --- | --- |
| **V27(cin - V27Ref)** | -0.03256972 | -0.01393028 | -10.734 | 0.009 |
| **V27(cin - V27Ref)** | -0.03256972 | -0.01393028 | -10.734 | 0.009 |

Table. S7 Significant values of Amide I, 1600-1690 cm^-1^ C=O stretching for frozen samples stored for a period of 4-week.

| **Pairs** | **95% confidence interval**  **Lower-Upper** | | **T-value** | **Two-side p-value** |
| --- | --- | --- | --- | --- |
| **V04(Cinn -Cumin)** | 0.02105333 | 0.03538800 | 6.319 | 0.024 |
| **V04(Cum-Cinn)** | -0.02105333 | -0.00671867 | -6.319 | 0.024 |
| **V04(Cinn-Cum)** | 0.00671867 | 0.03538800 | 6.319 | 0.024 |

Table. S8 Significant values of Amide II, 1480−1575 cm^-1^ CN stretching for frozen samples stored for a period of 1-week.

| **Pairs** | **95% confidence interval**  **Lower-Upper** | | **T-value** | **Two-side p-value** |
| --- | --- | --- | --- | --- |
| **V13(Vitc – Ref)** | 0.06103667 | 0.10076902 | 6.610 | 0.022 |
| **V13(Ging – Ref)** | 0.03402667 | 0.06220358 | 5.196 | 0.035 |
| **V13(Gar – Ref)** | 0.04172667 | 0.07820428 | 4.922 | 0.039 |
| **V13(Ref- Garl)** | -0.04172667 | -0.00524906 | -4.922 | 0.039 |
| **V13(Ref-VitC)** | -0.06103667 | -0.02130431 | -6.610 | 0.022 |

Table. S9 Significant values of Amide A, 3300cm^-1^ NH stretching for frozen samples stored for a period from 1-week to 4-weeks.

| **Pairs** | **95% confidence interval**  **Lower-Upper** | | **T-value** | **Two-side p-value** |
| --- | --- | --- | --- | --- |
| **V20Garl - V13Garl** | -0.26977689 | -0.02136311 | -5.043 | 0.037 |
| **V13Cur - V20Turm** | 0.05723517 | 0.08704483 | 20.825 | 0.002 |
| **V13VitC - V20VitC** | -0.02563241 | -0.01072759 | -10.496 | 0.009 |

Table. S10 Significant values of Amide I, 1600−1690 C=O stretching for frozen samples stored for a period from 1-week to 4-weeks.

| **Pairs** | **95% confidence interval**  **Lower-Upper** | | **T-value** | **Two-side p-value** |
| --- | --- | --- | --- | --- |
| **V20Ref - V04Ref** | -0.14255172 | -0.02158161 | -5.838 | 0.028 |
| **V20Turm - V04Turm** | -0.07167516 | -0.00462484 | -4.896 | 0.039 |
| **V13Cum - V20Cum** | 0.01762907 | 0.05280426 | 8.615 | 0.013 |
| **V13Vitc - V27 Vitc** | -0.07156777 | 0.00746777 | -3.490 | 0.073 |

Table. S11 Significant values of Amide II, 1480−1575 CN stretching, NH bending for frozen samples stored for a period from 1-week to 4-weeks.

| **Pairs** | **95% confidence interval**  **Lower-Upper** | | **T-value** | **Two-side p-value** |
| --- | --- | --- | --- | --- |
| **V13Ref - V04Ref** | -0.13419813 | 0.00102480 | -4.237 | 0.051 |
| **V13Cinn - V20Cinn** | 0.01002786 | 0.08377214 | 5.473 | 0.032 |
| **V04Cinn - V20Cinn** | -0.00547245 | 0.23387911 | 4.106 | 0.055 |
| **V13Ging - V20Ging** | 0.11574227 | 0.20303107 | 15.713 | 0.004 |
| **V04Ging - V20Ging** | 0.07050548 | 0.29585452 | 6.995 | 0.020 |
